# Estimation of Three-Dimensional Ground Reaction Force and Center of Pressure During Walking Using a Machine-Learning-Based Markerless Motion Capture System

**DOI:** 10.3390/bioengineering12060588

**Published:** 2025-05-29

**Authors:** Ru Feng, Ukadike Christopher Ugbolue, Chen Yang, Hui Liu

**Affiliations:** 1 School of Sports and Health, Nanjing Sport Institute, Nanjing 210014, China; fralida@163.com (R.F.); chen_yang@nsi.edu.cn (C.Y.); 2 School of Health and Life Sciences, University of the West of Scotland, South Lanarkshire, Hamilton G72 0LH, UK; u.ugbolue@uws.ac.uk; 3 School of Sport Science, Beijing Sport University, Beijing 100084, China

**Keywords:** ground reaction force, center of pressure, neural network, gait analysis, markerless motion capture

## Abstract

Objective: We developed two neural network models to estimate the three-dimensional ground reaction force (GRF) and center of pressure (COP) based on marker trajectories obtained from a markerless motion capture system. Methods: Gait data were collected using two cameras and three force plates. Each gait dataset contained kinematic data and kinetic data from the stance phase. A multi-layer perceptron (MLP) and convolutional neural network (CNN) were constructed to estimate each component of GRF and COP based on the three-dimensional trajectories of the markers. A total of 100 samples were randomly selected as the test set, and the estimation performance was evaluated using the correlation coefficient (r) and relative root mean square error (rRMSE). Results: The r-values for MLP in each GRF component ranged from 0.918 to 0.989, with rRMSEs between 5.06% and 12.08%. The r-values for CNN in each GRF component ranged from 0.956 to 0.988, with rRMSEs between 6.03–9.44%. For the COP estimation, the r-values for MLP ranged from 0.727 to 0.982, with rRMSEs between 6.43% and 27.64%, while the r-values for CNN ranged from 0.896 to 0.977, with rRMSEs between 6.41% and 7.90%. Conclusions: It is possible to estimate GRF and COP from markerless motion capture data. This approach provides an alternative method for measuring kinetic parameters without force plates during gait analysis.

## 1. Introduction

The common laboratory measurement scheme of gait analysis involves a combination of a marker = based motion capture system and force plates. This scheme is costly, requires trained experts to administer, and requires considerable time for set-up and processing, all of which preclude widespread adoption [1,2]. A marker-based motion capture system was used to analyze the kinematics characteristics of walking by tracking human motion [3]. The system used retro-reflective markers, making the process time-consuming and requiring highly specialized expertise. Additionally, the markers could hinder natural human movement [4]. A force plate was used to measure the magnitude and direction of the forces exerted on the plates by the person’s feet and body during walking. It could accurately identify the gait cycle and phases of walking [5]. However, the force plate measurements were only feasible under controlled conditions in specialized laboratory settings, and the continuous kinetic characteristics of gait could not be collected due to the limited number of force plates [6]. During testing, participants were required to step on one force plate with one foot, which was challenging for individuals with gait disorders.

In recent years, markerless motion capture systems based on computer vision and machine learning have been used to measure the kinematic characteristics of walking. Numerous studies have confirmed the accuracy and validity of these methods in gait analysis, supporting the application of markerless motion capture systems in this field [7,8,9,10,11]. Nakano [12] implemented markerless motion capture using OpenPose with multi-camera synchronization and compared the methods accuracy with a marker-based motion capture system. The error in recognizing human body markers was found to be 30 mm or less. Liu Hui [13] used the FastMove 3D Motion (Dalian Fast Move Technology Company, Dalian, Liaoning, China) to automatically identify the throwing movements of 12 javelin athletes, achieving a correlation coefficient greater than 0.98 between the three-dimensional coordinates of automatically and manually identified markers.

To address the challenge of measuring kinetic characteristics during walking, some studies have focused on estimating ground reaction force (GRF) using machine learning models. Various input parameters and machine learning algorithms have been employed, yielding high correlation coefficients and small errors between estimated values and actual values, confirming the feasibility of machine learning in GRF estimation [14,15,16,17,18,19,20,21]. However, existing studies still have certain limitations. The input parameters of the estimation models have primarily relied on the plantar pressure or kinematic information acquired from wearable devices [17,19,20,21,22,23]. Some studies have used kinematic parameters of human movements acquired through marker-based motion capture systems [15,24,25]. These approaches require placing multiple markers on the body or using inertial measurement sensors, which do not align with the goal of a portable gait analysis system. In addition, most studies have focused solely on estimating GRF without considering the center of pressure (COP), which is essential for calculating kinetic parameters such as joint moments [24]. Only a few research studies have attempted to estimate COP during walking, but they have used relatively small datasets and produced poor estimation results, particularly in the medial–lateral component of COP [17,18,26].

In summary, an ideal gait analysis system should be simplified and portable and should preferably avoid direct contact with the participants. Machine learning algorithms offer promising possibilities for markerless motion capture systems and kinetic parameter estimations. Therefore, the purpose of this study is to estimate GRF and COP during walking based on marker trajectories obtained from a markerless motion capture system. This study applies two neural network models, a multilayer perceptron (MLP) and a convolutional neural network (CNN), to achieve this estimation. We hypothesize that all models will achieve correlation coefficients greater than 0.9 and relative root mean square errors (rRMSE) below 10%. Furthermore, we hypothesize that the CNN model will outperform the MLP model, yielding higher correlation coefficients and lower rRMSE values. This study proposes to provide a solution for gait analysis without force plates and to contribute to the development of a widely accessible, portable gait analysis system in the future.

## 2. Materials and Methods

### 2.1. Participants

A total of 146 college students were recruited, including 62 males (age: 20.3 ± 1.2 years; height: 1.77 ± 0.06 m; weight: 71.8 ± 9.5 kg) and 84 females (age: 19.8 ± 1.4 years; height: 1.64 ± 0.06 m; weight: 56.0 ± 8.1 kg). All participants had no history of musculoskeletal disorders, and informed consent was obtained from all participants prior to starting the study. The use of human participants in this study was approved by the Internal Review Board.

### 2.2. Data Collection

**Figure 1:**
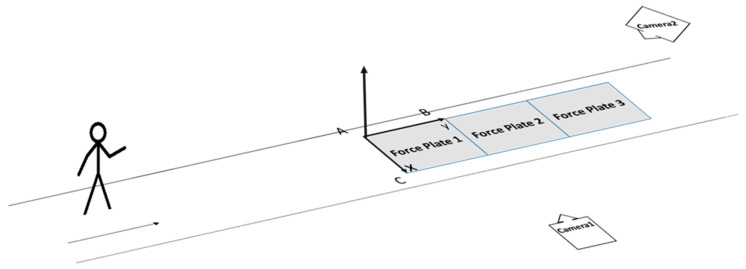
Test site of gait collection and spatial coordinate system diagram.

The layout of the test site is shown in Figure 1. The walking path was 8 m in length. Two high-speed cameras (FDR-AX700, Sony Group Corp, Tokyo, Japan) were positioned in front of and lateral to the participant to capture gait kinematics data at a sampling rate of 100 Hz.

Ground reaction force and center of pressure data were recorded at a sampling rate of 1000 Hz using three force plates (BMS400600, AMTI Inc., Watertown, MA, USA). Force Plate 1 and Force Plate 3 collected kinetic signals from the right foot, while Force Plate 2 collected kinetic signals from the left foot (Figure 1).

**Figure 2:**
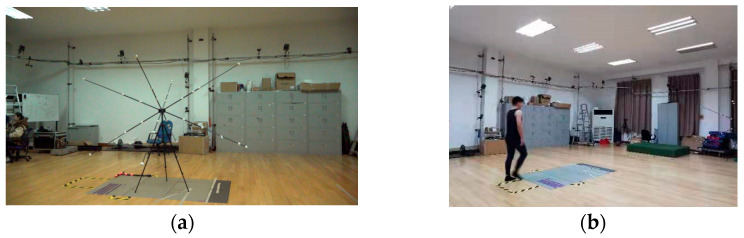
Calibration and testing site: (**a**) spatial calibration; (**b**) testing site.

A calibration frame and landmark were used to perform the three-dimensional calibration (Figure 2a). Participants were asked to walk barefoot at a self-generated speed. The participants were given sufficient training trials before the recording session. Gait data were collected 10 times for each subject (Figure 2b).

### 2.3. Data Processing

**Figure 3:**
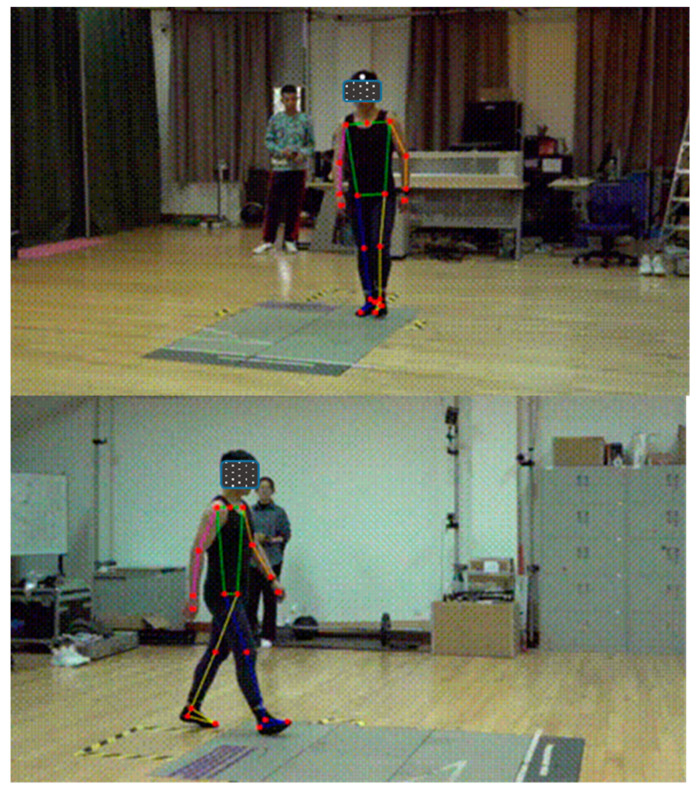
Pose estimation of whole body.

The walking video for each participant was edited into 10 clips, each containing a full gait cycle, using Adobe Premiere 2020 (Adobe Systems Incorporated, San Jose, CA, USA). Human pose estimation and three-dimensional data coordinates of 21 full-body markers were automatically obtained using the FastMove 3D Motion (Version 2.1.3, Dalian Fast Move Technology Company, Dalian, Liaoning, China) (Figure 3). Fastmove is a deep learning algorithm-based motion capture system that utilizes a deep convolutional neural network for human posture recognition, trained on the COCO dataset [27]. For biomechanical analysis, Fastmove further used more than 4000 sets of sports action videos, with human body points manually labelled by experienced researchers for training [13]. Fastmove also transformed the two-dimensional coordinate positions from two synchronized videos to three-dimensional coordinates based on the direct linear transform method.

To ensure that the dataset aligned with the input and output requirements of the estimation models, each gait cycle was segmented into three phases: the first support phase of the right foot, the first support phase of the left foot, and the second support phase of the right foot. The kinematic and kinetic data within each support phase were normalized to 100 time points using cubic spline interpolation, with 0% and 100% corresponding to heel strike and toe-off, respectively.

**Figure 4:**
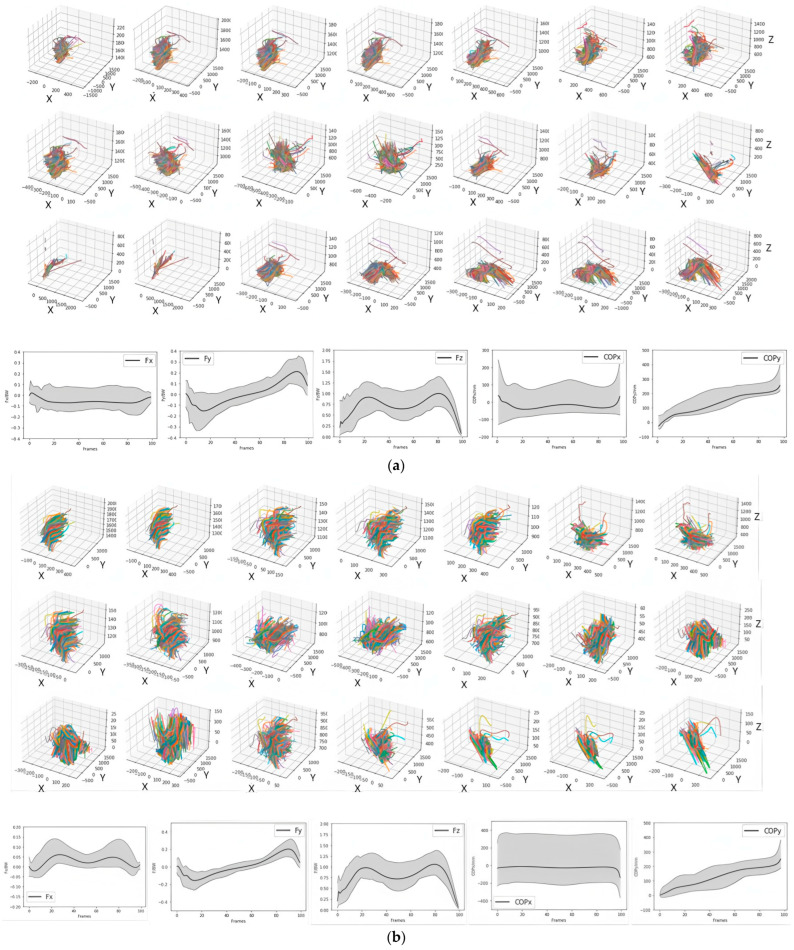
Input and output of gait dataset, including the three-dimensional (X, Y, Z) trajectories of 21 human full-body markers, the three-dimensional ground reaction forces (F_x_, F_y_, F_z_), and the two-dimensional positions of the center of pressure (COP_x_, COP_y_) from all the subjects. Different colors represent different samples of each subjects. (**a**) Dataset of right foot stance phase; (**b**) dataset of left foot stance phase.

Ground reaction force (GRF) was normalized to body weight (BW), while the center of pressure (COP) was analyzed in millimeters. The input for the estimation model consisted of the three-dimensional trajectories of 21 markers during a support phase (3 × 21 × 100), while the output was a single component of GRF or COP for the same support phase (1 × 100) (Figure 4).

### 2.4. Model Construction

**Figure 5:**
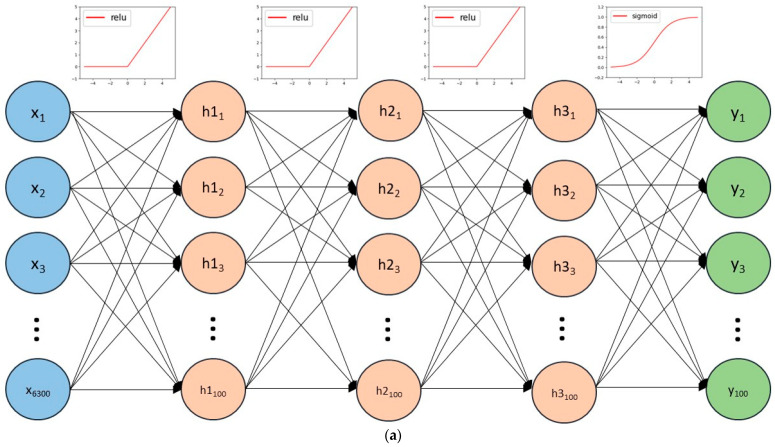

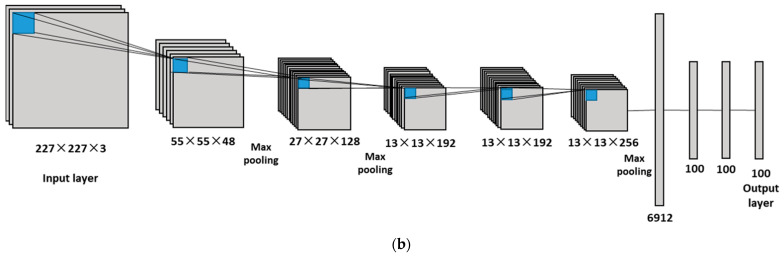
Model network structure of MLP and CNN: (**a**) model network structure of MLP; (**b**) model network structure of CNN.

We applied MLP and convolutional neural networks to estimate GRF and COP. The overall structure of the MLP used in this study is shown in Figure 5a. The input layers contained 6300 neurons, which were expanded into a one-dimensional vector by the parameters of kinematics. The hidden layer consisted of three layers, each containing 100 neurons, with the relu function used as the activation function between each hidden layer. The output layers contained 100 neurons, corresponding to a single component of GRF or COP in a time series. The activation function for the output layer was defined as a sigmoid function, and the loss function was defined by the root mean square error.

The second neural network model was a CNN, specifically utilizing AlexNet as the model structure (Figure 5b). AlexNet consisted of 8 layers, in which the first 5 layers were convolutional layers followed by 3 full connectional layers. To conform to the input format of the model, a cubic spline interpolation was used to interpolate the two-dimensional matrix of 21 marker trajectories into a 227 × 227 two-dimensional matrix. The coordinates (x, y, z) of each point were, respectively, mapped into the image additive colors (R, G, B).

When training the model, the number of the batch size selected for one training round was set to 128, and the epoch was set to 1000. The mean squared error (MSE) was selected as the loss function. For updating the weights and biases in the neural network, the stochastic gradient descent (SGD) optimizer was used for MLP, and the Adam optimizer was used for CNN.

Data processing and model construction were realized via Python 3.8 (Python Software Foundation, Amsterdam, The Netherlands). Data preprocessing was realized through Pandas and NumPy. The interpolation of the three-dimensional trajectories of the input parameters and the generation of RGB pictures were realized through Scipy (www.scipy.org) and OpenCV (www.opencv.org). The construction and training of each learning model was realized through Tensorflow (https://tensorflow.google.cn/).

### 2.5. Model Assessment

In order to assess the estimation performance of the model, the gait data of 100 samples from 5 males and 5 females were randomly selected as test sets, and the rest of the gait data were selected in the training sets (Table 1).

For each model, the relative root mean square error (rRMSE) and correlation coefficient (r) were used to evaluate the estimation accuracy.

(1) $$rRMSE=\frac{RMSE}{\frac{1}{2}\left[\sum_{i=1}^{2}\left[\max\left(u_{i}\left(t\right)\right)-\min\left(u_{i}\left(t\right)\right)\right]\right]}$$

(2) $$r=\frac{\sum_{t=1}^{T}\left(u_{1}\left(t\right)-{\bar{u}}_{1}\right)\left(u_{2}\left(t\right)-{\bar{u}}_{2}\right)}{\sqrt{\sum_{t=1}^{T}{\left(u_{1}\left(t\right)-{\bar{u}}_{1}\right)}^{2}}\sqrt{\sum_{t=1}^{T}{\left(u_{2}\left(t\right)-{\bar{u}}_{1}\right)}^{2}}}$$

where $u_{1}\left(t\right)$ is the estimated value in each frame and $u_{1}\left(t\right)$ is the true value in each frame.

### 2.6. Statistical Methods

A paired-samples T-test was used to compare the rRMSE and r-value of the two neural network models for each component of GRF and COP. Statistical significance was defined as *p* < 0.05, and all statistical analyses were realized through Statsmodels (www.statsmodels.org).

## 3. Results

**Figure 6:**
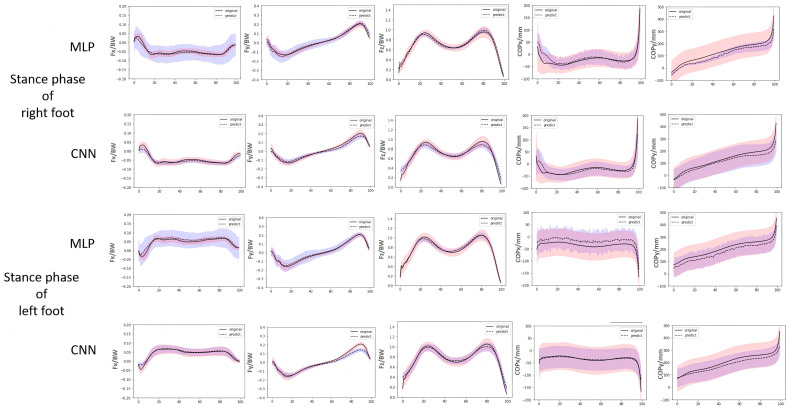
The curves of each component of GRF and COP predicted by CNN and MLP.

The figures below show the curves of each component across 100 test samples. The solid line represents the true average curve, while the dashed line represents the estimated average curve (Figure 6). In the figures and tables, x, y, and z correspond to the medial–lateral, anterior–posterior and vertical components, respectively.

The r-value for the medial–lateral component of GRF estimated by CNN was significantly higher than those estimated by MLP in both the right (*p* < 0.001) and left stance phases (*p* < 0.001) (Table 2). The rRMSE estimated by CNN was significantly lower than those estimated by MLP in both the right (*p* < 0.001) and left stance phases (*p* < 0.001) (Table 3).

The r-value of the anterior–posterior component of GRF estimated by CNN was significantly higher than that estimated by MLP in the right stance phase (*p* = 0.019) (Table 2). The rRMSE estimated by CNN was significantly lower than those estimated by MLP in the left stance phase (*p* < 0.001) (Table 3).

The r-value of the vertical component of GRF estimated by CNN was significantly higher than those estimated by MLP in the left stance phase (*p* < 0.001) (Table 2). The rRMSE estimated by CNN was significantly lower than that estimated by MLP in the left stance phase (*p* < 0.001) (Table 3).

The r-value of the medial–lateral component of COP estimated by MLP was significantly higher than that estimated by CNN in the left stance phase (*p* < 0.001) (Table 2). The rRMSE of the medial–lateral component of COP estimated by MLP was significantly lower than those estimated by CNN in both the right (*p* = 0.026) and left stance phases (*p* < 0.001) (Table 3).

The r-value of the anterior–posterior component of center of pressure estimated by CNN was significantly higher than that estimated by MLP in the left stance phase (*p* < 0.001) (Table 2). The rRMSE estimated by MLP was significantly lower than that estimated by CNN in the right stance phase (*p* < 0.001) (Table 3).

## 4. Discussion

In our study, two machine learning algorithms, MLP and CNN, were used to construct estimation models of GRF and COP based on a markerless motion capture system. A total of 90% of the models (18 out of 20) of the correlation coefficients between the estimated and true values exceeded 0.9, and 95% of the models (19 in 20) of the relative root mean square errors were below 15%. Predictions were considered excellent if the coefficient of cross-correlation was greater than 0.9 and the relative RMS error was smaller than 15% [28]. The results demonstrate the feasibility of estimating the kinetic parameters based on the kinematic data acquired by the markerless motion capture system, and the system provides a potential alternative for GRF and COP measurements without force plates in gait analysis.

We first estimated the three-dimensional GRF during the stance phase of walking. Our average r-values for GRF, ranging from 0.918 to 0.967 for the medial–lateral direction, 0.984 to 0.989 for anterior–posterior direction, and 0.966 to 0.978 for the vertical direction, were slightly higher than previously reported values (0.64–0.71 for the medial–lateral, 0.92–0.96 for the anterior–posterior, and 0.94–0.97 for the vertical directions) in a walking task [17,18,29]. In previous GRF estimation studies, the input parameters were plantar pressure data collected from pressure sensors [30,31,32,33] and kinematic data collected from inertial sensors [21,34] and accelerometers [16,19]. Only a few studies have used trajectories of human markers [15,25] or kinematic data of human joints [35] based on a marker-based motion capture system for GRF estimation. Johnson [15] applied a similar machine learning algorithm with comparable input parameters, but the movement differed from our study. Their CNN correlation coefficients in the estimation of the vertical GRF component were more than 0.992, and the rRMSE values were less than 4.16%. The accuracy of our study was slightly lower than that of previous studies, which may have been due to the reduced precision of kinematic data obtained from the markerless motion capture system.

We also estimated the two components of COP during the stance phase of walking. Except for the medial–lateral component of COP in the left stance phase estimated by MLP, all rRMSE values of the estimated COP in the models were less than 10%. Accurate COP estimation is essential for inverse dynamics, making research in this area particularly valuable. Lee and Park [18] estimated COP with a single inertial measurement unit. They reported an rRMSE of 19.54% for the medial–lateral component of COP and 8.22% for the anterior–posterior component. In another study, an MLP model was constructed to estimate the COP during walking based on kinematic parameters collected using a marker-based motion capture system [17]. The r-value of the medial–lateral component of COP reported was 0.37, and the RMSE was 10.6 mm. The r-value of the anterior–posterior component was 0.96, and the RMSE was 9.2 mm. Similar to previous findings, our study achieved a higher estimation accuracy with respect to the anterior–posterior component of COP when compared to the medial–lateral component; the high variability in the medial–lateral component of COP during walking could have affected the low estimation accuracy [36].

The choice of the optimal machine learning algorithm varied depending on the GRF and COP component being estimated. Both MLP and CNN have been widely used in previous GRF estimation studies, but there is no clear consensus on which algorithm performs better [6,15,24]. Due to differences in experimental settings, measuring equipment, the amount and type of data, it was challenging to select the most appropriate algorithm as the model for the current study.

Our results indicate that the estimation accuracy varied across the different GRF and COP components. The anterior–posterior component of GRF showed the highest correlation with true values, whereas the medial–lateral component of COP showed the lowest correlation. These results align with previous research suggesting that human movement has a significant effect on the anterior–posterior component of GRF [24]. In contrast, the low accuracy of the medial–lateral COP component may be attributed to its relatively small magnitude compared to overall body movement fluctuations.

Despite the increasing interest in markerless motion capture, marker-based systems are still preferred for clinical gait analysis [37]. This is actually related to the application purpose of GRF estimation, where GRF estimation research aims to enable kinetic analysis under non-laboratory conditions in the future. If the kinematic data acquired by marker-based systems are used as the estimation input of GRF, a motion capture system under laboratory conditions is still needed. This study constructed GRF and COP estimation models using three-dimensional trajectories of markers collected from a markerless motion capture system, highlighting its potential for gait analysis under non-laboratory conditions in the future. These findings lay the foundation for gait analysis outside of laboratory settings.

While the results of this study provide a novel approach for estimating GRF and COP during walking, several limitations should be acknowledged. First, all the subjects were college students. The datasets only contained healthy young participants’ gait data, meaning the models may not accurately estimate kinetics data of participants with dysfunction. This problem could be partially solved by adding additional subjects of different ages and genders and with various dysfunctions in future studies. Second, due to the exploratory nature of this study, we only considered two machine learning algorithms, which may have constrained the estimation accuracy.

## 5. Conclusions

In this study, a large gait dataset containing 21 marker trajectories and the full ground reaction force was constructed using a markerless motion capture system and force plates.

This study developed models to estimate the three-dimensional GRF and COP of walking movements based on full-body marker trajectories obtained from a markerless motion capture system. Most of the models achieved good estimation accuracy, where the rRMSE was less than 15% for walking. The estimation of GRF and COP from markerless motion capture data is not only achievable but also presents a promising alternative for measuring kinetic parameters in gait analysis without force plates.

CNN appeared to outperform MLP in estimating the medial–lateral components of GRF and COP during walking.

## Figures and Tables

**Table 1:** Test sets and training sets in each stance phase.

Stance Phase	Training Sets		Test Sets	
	Subjects	Samples	Subjects	Samples
Right	135	2710	10	100
Left	135	1305	10	100

**Table 2:** The correlation coefficient of each component of GRF and COP by MLP and CNN.

Stance Phase	Component	MLP	CNN	T-Value	*p*-Value
Right	GRFx	0.918 ± 0.034	0.956 ± 0.018	49.370	**<0.001**
	GRFy	0.984 ± 0.008	0.987 ± 0.004	5.693	**0.019**
	GRFz	0.971 ± 0.012	0.975 ± 0.012	3.247	0.074
	COPx	0.901 ± 0.137	0.896 ± 0.054	0.056	0.813
	COPy	0.978 ± 0.015	0.974 ± 0.016	2.356	0.128
Left	GRFx	0.920 ± 0.030	0.967 ± 0.012	104.620	**<0.001**
	GRFy	0.989 ± 0.004	0.988 ± 0.004	1.499	0.223
	GRFz	0.966 ± 0.019	0.978 ± 0.009	17.185	**<0.001**
	COPx	0.727 ± 0.163	0.924 ± 0.033	70.797	**<0.001**
	COPy	0.982 ± 0.009	0.977 ± 0.011	6.614	**<0.001**

The bold font indicates significant differences (*p* < 0.05).

**Table 3:** The rRMSE of GRF and COP components estimated by MLP and CNN (%).

Stance Phase	Component	MLP	CNN	T-Value	*p*-Value
Right	GRFx	12.08 ± 1.49	9.44 ± 1.39	83.777	**<0.001**
	GRFy	6.23 ± 1.42	6.49 ± 0.66	1.413	0.237
	GRFz	7.06 ± 1.04	7.37 ± 0.85	2.573	0.112
	COPx	9.33 ± 3.47	7.9 ± 2.83	5.090	**0.026**
	COPy	8.28 ± 1.95	6.81 ± 1.32	19.408	**<0.001**
Left	GRFx	11.05 ± 1.34	7.29 ± 1.17	222.221	**<0.001**
	GRFy	5.06 ± 0.68	8.03 ± 0.89	353.561	**<0.001**
	GRFz	7.71 ± 1.39	6.03 ± 0.75	56.655	**<0.001**
	COPx	27.64 ± 6.12	6.41 ± 1.44	570.769	**<0.001**
	COPy	6.43 ± 1.8	6.52 ± 1.19	0.076	0.783

The bold font indicates significant differences (*p* < 0.05).

## Data Availability

The original contributions presented in this study are included in the article. Further inquiries can be directed to the corresponding author.

## Abbreviations

The following abbreviations are used in this manuscript:

GRF	Ground reaction force
COP	Center of pressure
MLP	Multi-layer perceptron
CNN	Convolutional neural network
rRMSE	Relative root mean square errors
